# Examining Miliary Disease Etiology in a *Coccidioides*-Endemic Center: A Retrospective Cohort Study

**DOI:** 10.3390/jof10010029

**Published:** 2023-12-31

**Authors:** Ashley M. Scott, James Ray Lim, Reubender Randhawa, Jason Lee, Kavitha Yaddanapudi, Brooke Rabe, Joshua Malo

**Affiliations:** 1 Department of Pulmonary and Critical Care Medicine, University of Arizona, Tucson, AZ 85721, USA; jmalo@arizona.edu; 2 Department of Infectious Disease, University of Arizona, Tucson, AZ 85721, USA; limjm1@gmail.com; 3 Department of Internal Medicine, University of Arizona, Tucson, AZ 85721, USA; randhawa.md@outlook.com; 4 Department of Radiology, University of Arizona, Tucson, AZ 85721, USA; jasonlee1@arizona.edu (J.L.); yaddanapudi@radiology.arizona.edu (K.Y.); 5 Department of Biostatistics, Bio5 Institute, University of Arizona, Tucson, AZ 85721, USA

**Keywords:** miliary, disseminated, metastatic, coccidioidomycosis, *Coccidioides*, valley fever, tuberculosis, TB, cancer

## Abstract

**Background:** A miliary pattern on chest imaging is often attributed to tuberculosis (TB) infection. However, a myriad of conditions can cause a miliary pattern, many of which are imminently life-threatening. **Research Question:** The primary aim of our study is to elucidate the potential causes of miliary chest imaging patterns to improve workup and empiric therapy selection. The secondary aims are to discern the predictors of miliary disease etiology and to determine whether appropriate empiric antimicrobial therapies were given. **Study Design and Methods:** In this retrospective cohort study, we searched a radiology database for patients with chest imaging studies described by the word “miliary”. Subjects were excluded if they were under 18 years of age and if there were insufficient objective data to support a miliary disease etiology. A radiologist independently reviewed all imaging studies, and studies that did not appear to have a true miliary pattern were excluded. The collected data include patient demographics, immunocompromising risk factors, conditions associated with miliary disease, β-D-glucan levels, serum eosinophil count, and empiric therapies received. **Results:** From our 41-patient cohort, 22 patients (53.7%) were clinically diagnosed with coccidioidomycosis, 8 (19.5%) with TB, 7 (17.1%) with metastatic solid cancer, 1 (2.4%) with lymphoma, 1 (2.4%) with other (*Mycobacterium simiae*), and 3 (7.3%) with unknown diseases (the sum equals 42 patients because one individual was diagnosed with both coccidioidomycosis and TB). All six patients with greater than 500 eosinophils/μL were diagnosed with coccidioidomycosis. Of the 22 patients diagnosed with coccidioidomycosis, 20 (90.91%) were empirically treated with an antifungal regimen. Of the eight patients with TB, six were empirically treated for TB. **Interpretation:** Based on our data from a *Coccidioides*-endemic region with close proximity to tuberculosis-endemic areas, the leading cause of miliary disease is coccidioidomycosis, although TB and cancer are also common etiologies. Serum eosinophilia and elevated β-D-glucan levels were strongly predictive of coccidioidomycosis in our patient cohort with a miliary chest imaging pattern.

## 1. Introduction

A miliary pattern on chest imaging is usually indicative of a life-threatening, disseminated disease process. A miliary pattern is defined by numerous nodules, 1–3 millimeters in size, randomly distributed throughout the lungs. While a miliary pattern can be indicative of disseminated tuberculosis (TB), it is not pathognomonic for TB.

Various metastatic cancers and other neoplastic processes are known causes of miliary disease [1,2,3,4,5,6,7,8,9,10,11,12,13,14,15,16,17,18,19,20]. Several interstitial lung diseases can present atypically with a miliary pattern, such as sarcoidosis [21], pulmonary alveolar microlithiasis [22], various pneumoconioses [23], hypersensitivity pneumonitis [24], lipoid pneumonia [25], vaping-associated lung injury [26], drug-induced pneumonitis [27], and organizing pneumonia [28]. Miscellaneous causes of miliary disease include multifocal micronodular pneumocyte hyperplasia [29] and pulmonary hemosiderosis due to mitral stenosis [30]. Various life-threatening infections aside from TB can also manifest with a miliary pattern on chest imaging. Endemic mycoses, such as coccidioidomycosis, are known causes of miliary disease [31,32,33,34,35], as are other fungal infections [36,37,38,39]. Several bacterial infections, such as non-tuberculous mycobacteria [40], actinomycosis [41], and various other types of infection [42,43], can present with a miliary pattern, as can viral [44,45] and parasitic [46] infections.

In an observational study at the University of Texas in Houston that involved 53 patients with miliary nodules, tuberculosis was diagnosed in 28.3% of patients, sarcoidosis in 22.6%, silicosis in 13.2%, extrathoracic malignancy in 9.4%, and histoplasmosis in 7.6%; *Pneumocystis jirovicii* pneumonia, *Mycobacterium avium* complex, Epstein–Barr infection, cryptococcosis, aspergillosis, and primary lung cancer were diagnosed in one case each [47]. (It is worth noting that Houston is not in a *Coccidioides*-endemic region.) A Greek retrospective review of eight cases of miliary patterns on high-resolution computed tomographic imaging attributed the causes to tuberculosis (three), *Candida albicans* (one), sarcoidosis (three), and metastatic adenocarcinoma (one) [48].

We performed a retrospective observational study of patients with miliary chest imaging patterns at a large academic center in *Coccidioides*-endemic Tucson, Arizona, USA, which is located 70 miles from Sonora, Mexico. The primary aim of our study is to determine the type and prevalence of diseases associated with miliary chest imaging findings. The secondary aims are to discern the predictors of miliary disease etiology and to determine whether appropriate empiric antimicrobial therapies were given to patients with a miliary disease pattern on chest imaging.

## 2. Methods

### 2.1. Data Collection

**Figure 1:**
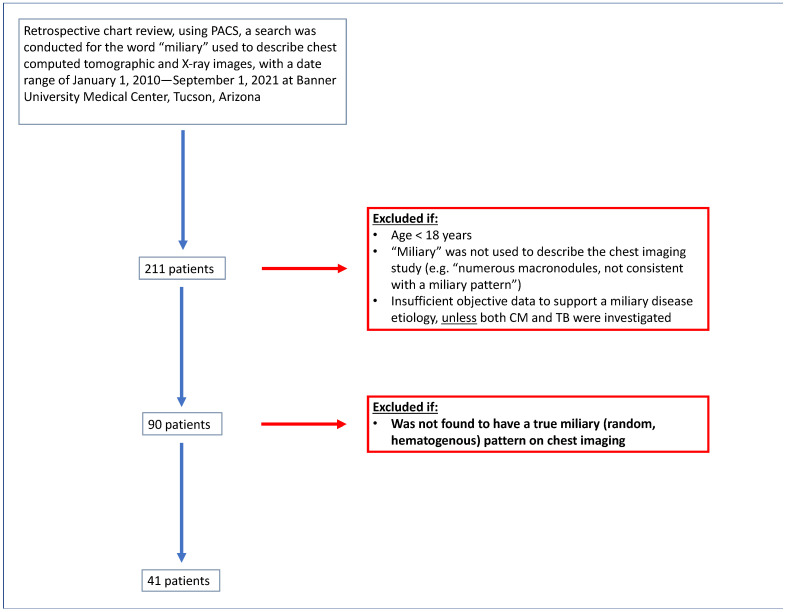
Overview of patient selection.

This is a single-center retrospective cohort study from the University of Arizona, a large tertiary academic center in Tucson, Arizona, USA. The study received institutional review board (IRB)-exempt status (IRB 2108172376). Using the radiology picture archiving and communication systems (PACS), a search was conducted for the word “miliary” used to describe chest computed tomographic (CT) and chest radiographic images, with a date range of 1 January 2010–1 September 2021, yielding a 211-patient list. We excluded patients who did not meet the following criteria: age ≥ 18 years and “miliary” as a descriptor of the chest imaging study (e.g., if the imaging study was found by the search term “miliary” due to the description, “non-miliary”). Patients were further excluded if no potential etiology for the miliary disease was discovered unless investigations for both coccidioidal infection and TB were performed and negative. A total of 90 patients remained after those exclusion criteria were applied (see Figure 1 for a flow chart of our study design).

The collected data include patient demographics (age, gender, and race); pre-existing diagnoses that are known causes of miliary disease; the presence of immunocompromising factors (specifically, we collected data on the presence of diabetes, human immunodeficiency virus (HIV), untreated HIV, HIV with cluster of differentiation 4 (CD4) count <200 cells/mm^3^, HIV with CD4 count ≥200 cells/mm^3^, cancer, and cirrhosis; the use of chemotherapy, immunotherapy, prednisone ≥20 mg/day, biologic response modifiers, and antimetabolites; and solid-organ or bone marrow transplant status); absolute eosinophil count; 1,3-β-D-glucan value; data in support of diagnoses associated with miliary disease (pathology results; the results of *Coccidioides* serologies (enzyme immunoassay for the detection of immunoglobulin M (IgM) and immunoglobulin G (IgG) antibodies; immunodiffusion assay against IgM (tube precipitin) and IgG (complement fixation); and complement fixation titers of antibodies directed against *Coccidioides*); microbiologic cultures; the results of interferon-γ release assay for TB; the results of tuberculin skin testing; acid-fast bacilli smear and culture results; and the results of TB polymerase chain reaction testing); and empiric therapies (antifungals directed against coccidioidal infection (more specifically, amphotericin or azole antifungal therapy) and the receipt of antituberculous therapy (it was specified whether the routine treatment of rifampin, isoniazide, pyrazinamide, and ethambutol (RIPE); another regimen; or RIPE plus adjunctive therapies was given)).

The clinical diagnosis of coccidioidomycosis was made based on positive coccidioidal IgM or IgG serologies, cultures, or histopathology. The clinical diagnosis of tuberculosis was made based on a positive result obtained from cultures, pathology, TB polymerase chain reaction, tuberculin skin testing, or interferon-γ release assay for TB.

### 2.2. Radiological Verification

A cardiothoracic-trained radiologist reviewed the imaging studies. Patients were excluded if they did not have a true random hematogenous miliary imaging pattern. The miliary pattern, per the Fleischner Society definition, was characterized by the presence of micronodules measuring less than 3 mm. These nodules exhibited a uniform distribution in a random pattern, without peri-lymphatic, centrilobular, or geographic preference. This yielded a 41-patient cohort (see Figure 1 for a flow chart of our study design).

### 2.3. Statistical Analysis

Descriptive statistics were employed to illustrate the differences between patients with missing β-D-glucan and eosinophil data and those with complete data.

Logistic regression models were used to assess the relationship between a diagnosis of coccidioidomycosis and three variables: β-D-glucan level, eosinophil count, and percent eosinophils. The β-D-glucan level was analyzed both as a continuous variable and as a binary variable using a threshold of 80 pg/mL, following the manufacturer’s guidance. Eosinophil variables were treated as continuous. No additional variables were included due to the limited sample size.

Model performance was evaluated by the area under the receiver operating characteristic curve (AUC) and the prediction accuracy was assessed via leave-one-out cross-validation. Bootstrap resampling with 2000 stratified replicates was used to obtain 95% confidence intervals for the AUC.

We selected the predictive model with the highest accuracy to estimate the mean probability of coccidioidomycosis among patients with elevated β-D-glucan levels of 150 pg/mL.

Barnard’s exact test was used to examine the association between a diagnosis of coccidioidomycosis and elevated eosinophil counts or percentages.

The potential confounding effect of age on the relationship between the β-D-glucan level, eosinophilia, and diagnosis was assessed using multiple linear regression models. Prediction modeling and statistical testing were performed using R version 4.2.2.

## 3. Results

The average age of the cohort was 51.2 years, with 29 (70.7%) males and 12 (29.3%) females. Non-Hispanic whites comprised the plurality with 17 patients (41.5%), followed by 13 (31.7%) LatinX patients, 4 (9.8%) Native Americans, 4 (9.8%) Asians and Pacific Islanders, 2 (2.4%) Blacks, and 2 (4.9%) unknown.

**Figure 2:**
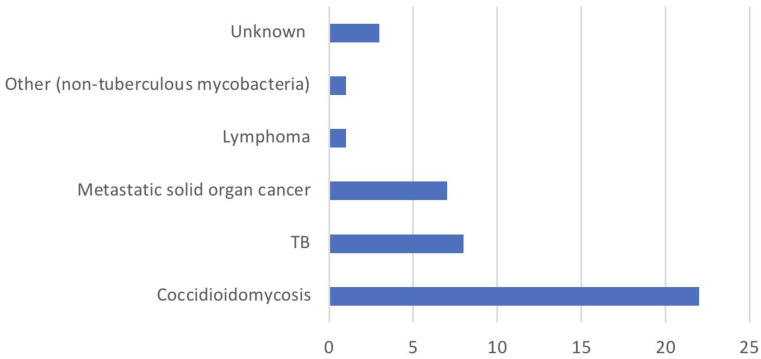
Diagnoses associated with miliary disease.

In our 41-patient cohort, 22 patients (53.7%) were clinically diagnosed with coccidioidomycosis, 8 (19.5%) with TB, 7 (17.1%) with metastatic solid cancer, 1 (2.4%) with lymphoma, 1 (2.4%) with other (*Mycobacterium simiae*), and 3 (7.3%) with unknown disease processes (see Figure 2). See Supplemental Table S1 for the variables used to assist in the clinical diagnoses of coccidioidomycosis and TB. It is worth noting that the sum adds up to 42 patients, as one unfortunate individual was diagnosed with both coccidioidomycosis and TB (see Table 1).

Of the 22 patients with coccidioidomycosis, 20 (90.91%) were empirically treated with some form of antifungal. Three patients were treated with amphotericin, and nineteen were treated with an azole, with two treated with both amphotericin and an azole. Of the eight patients with TB, six (75%) were empirically treated for TB. Four were treated with expanded antituberculous coverage. Of the four patients who received amphotericin, three had coccidioidomycosis, and one had TB (without coccidioidal infection). Of the 21 patients who empirically received an azole, 19 were diagnosed with coccidioidomycosis and 2 were not, both of whom were diagnosed with TB. Nine patients empirically received therapy for TB; of those, six patients were later diagnosed with TB, two with coccidioidomycosis, and one with non-tuberculous mycobacterial infection.

The metastatic malignancies that were diagnosed included pulmonary adenocarcinoma (three), a high-grade serous adenocarcinoma of Mullerian primary (one), renal cell carcinoma (one), papillary thyroid cancer (one), and hepatocellular carcinoma (one).

Of the eight patients with HIV and a CD4 count less than 200 cells/mm^3^, six (75%) were ultimately diagnosed with coccidioidomycosis, and two (25%) were diagnosed with TB (see Table 1). All five patients with cirrhosis (100%) were diagnosed with coccidioidomycosis, with one patient (20%) also diagnosed with TB (i.e., one unfortunate individual appeared to have both coccidioidomycosis and TB). Six of the nine patients with diabetes were diagnosed with coccidioidomycosis (67%), one with TB, one with metastatic solid organ cancer, and one with disseminated nontuberculous mycobacteria. All six patients with serum eosinophil counts above 500 eosinophils/μL were diagnosed with coccidioidomycosis.

Three logistic regression models were examined to assess the relationship between coccidioidomycosis, eosinophil count and percentage, and serum β-D-glucan levels. Eosinophil counts were available for 25 patients, and 22 patients had serum β-D-glucan levels. The five β-D-glucan levels below and eight above the detection limits were imputed with nearest integer values. Of these, the model including β-D-glucan as a continuous variable alone (model 1) yielded the highest predictive accuracy and an odds ratio significantly greater than 1 (1.006 95% CI [1.000, 1.012]). Other predictive models with dichotomized β-D-glucan levels and percent eosinophils were examined, but they were not notably different than those presented in Table 2. Using model 1, we estimated that the mean probability of a coccidioidomycosis diagnosis in patients with a β-D-glucan value of 150 pg/mL was 0.554 (95% CI [0.296, 0.785]).

The patient characteristics in terms of the status of β-D-glucan levels and eosinophils were similar across gender, age, race/ethnicity, diabetes status, and the presence of active cancer (see Supplemental Table S2). However, a final diagnosis of coccidioidomycosis (cocci.) was more prevalent in the group with observed data.

Serum eosinophil counts were available for 25 patients (see Table 3). Serum eosinophil count was elevated above 0.5 × 10^9^/L in six individuals, all of whom were diagnosed with coccidioidomycosis. Barnard’s exact test suggested associations between the diagnosis of coccidioidomycosis and elevated eosinophil count (>0.5 × 10^9^/L), with a *p*-value of 0.02.

Linear models of β-D-glucan levels and eosinophils regressed against age and diagnosis, and their interaction did not show evidence of age effects.

## 4. Discussion

To our knowledge, this is the first study to examine diagnoses associated with miliary chest imaging patterns in a *Coccidioides*-endemic region that also closely borders a TB-endemic region. The incidence of TB in the state of Sonora is 32.9 cases per 100,000 (the highest in Mexico), compared to 1.1 cases per 100,000 people in bordering Arizona [49] or 2.5 cases per 100,000 in the United States at large [50].

The leading diagnosis associated with miliary disease in our southern Arizona healthcare center was coccidioidomycosis (53.7%), followed by TB (19.5%), and metastatic solid cancer (17.1%). These represent treatable diseases that are imminently life-threatening in the face of diagnostic delay. Most patients who were ultimately diagnosed with coccidioidomycosis were empirically treated with an antifungal regimen (90.91%), while 75% of patients ultimately diagnosed with TB were empirically treated with an antituberculous regimen.

Elevated eosinophil count and β-D-glucan level are strongly associated with the diagnosis of coccidioidomycosis. All six patients with serum eosinophil counts above 500/μL were diagnosed with coccidioidomycosis. However, a significant proportion of patients, about half with low eosinophil counts and a third with low β-D-glucan levels, were also diagnosed with coccidioidomycosis. This finding suggests a considerable degree of uncertainty surrounding the predictive value of these tests in their lower ranges, underscoring the need for maintaining a high index of suspicion for coccidioidomycosis in the appropriate clinical context.

In our single-center, southern Arizona retrospective cohort, coccidioidomycosis, TB, and metastatic solid cancer appear to be the most common causes of miliary patterns on chest imaging. These represent treatable diseases that are imminently life-threatening in the face of diagnostic delay.

The results of this study should be interpreted with caution due to the limited sample size. Other limitations include the retrospective nature of this review and dependence on diagnostic studies ordered by other providers. Furthermore, the intention of this study was to specifically examine this cohort in an area where *Coccidioides* is endemic. As such, the results may not apply to miliary disease outside of these areas, although maintaining awareness of fungal pneumonia is beneficial in any setting.

Factors influencing the initial decision to test for β-D-glucan levels and eosinophils remain unknown. Understanding these factors is crucial, as they can introduce additional biases and confounding variables that may influence the observed associations. Therefore, the applicability of these findings should be further investigated in larger, more representative populations, and the decision-making process leading to the initial testing warrants further study.

A strength of our study is the multidisciplinary representation of pulmonary and critical care medicine, infectious disease, and radiology specialists in its design and evaluation. Our study’s location in a *Coccidioides*-endemic region also increases the validity of coccidioidal test interpretation.

## 5. Conclusions

In summary, while miliary disease is often attributed to TB, we found that coccidioidomycosis is an important etiology of miliary disease in *Coccidioides*-endemic regions. While this study suggests a potential role for eosinophil count and β-D-glucan levels in predicting coccidioidomycosis among patients with miliary lung disease, the large degree of uncertainty and potential biases underscore the need for further research and, importantly, that coccidioidomycosis should still be considered in patients with non-elevated eosinophil counts or β-D-glucan levels. A careful interpretation and application of these findings are advised until additional data become available. Coccidioidomycosis should be considered as a cause of miliary disease, given the severity of miliary coccidioidomycosis and the potential for poor outcomes. The initiation of empiric therapy should be considered in the appropriate clinical setting. This will likely become more salient, given the growing endemic range of *Coccidioides* in the setting of global climate change.

### Take Home Points

- Study question: What are the most common diagnoses associated with miliary chest imaging patterns in our southern Arizona center?
- Results: From our 41-patient cohort, 22 patients (53.7%) were clinically diagnosed with coccidioidomycosis, 8 (19.5%) with tuberculosis, 7 (17.1%) with metastatic solid cancer, 1 (2.4%) with lymphoma, 1 (2.4%) with other (*Mycobacterium simiae*), and 3 (7.3%) with unknown (the sum equals 42 patients because one unfortunate individual was diagnosed with both coccidioidomycosis and TB).
- Interpretation: Diseases associated with miliary chest imaging patterns are imminently life-threatening in the face of diagnostic delay.

## Figures and Tables

**Table 1:** Patient characteristics.

P								
	Cocci. (N = 21)	TB (N = 7)	Cocci. and TB (N = 1)	Metastatic Solid Tumor (N = 7)	Lymphoma (N = 1)	Other (N = 1)	Unknown (N = 3)	Total (N = 41)
Age								
Mean (SD)	48.905 (13.341)	43.875 (20.753)	36 (NA)	56.571 (13.365)	65.000 (NA)	59.000 (NA)	67.000 (6.083)	51.195 (15.381)
Sex								
Male	16 (76.2%)	6 (85.7%)	1 (100%)	3 (42.9%)	1 (100.0%)	1 (100.0%)	1 (33.3%)	29 (70.7%)
Female	5 (23.8%)	1 (14.3%)	0 (0.0%)	4 (57.1%)	0 (0.0%)	0 (0.0%)	2 (66.7%)	12 (29.3%)
Race/Ethnicity								
Non-Hispanic White	8 (38.1%)	1 (14.3%)	1 (100%)	4 (57.1%)	1 (100.0%)	0 (0.0%)	2 (66.7%)	17 (41.5%)
Black or African American	1 (4.8%)	0 (0.0%)	0 (0.0%)	0 (0.0%)	0 (0.0%)	0 (0.0%)	0 (0.0%)	1 (2.4%)
Asian or Pacific Islander	1 (4.8%)	1 (14.3%)	0 (0.0%)	2 (28.6%)	0 (0.0%)	0 (0.0%)	0 (0.0%)	4 (9.8%)
Hispanic	8 (38.1%)	3 (42.9%)	0 (0.0%)	0 (0.0%)	0 (0.0%)	1 (100.0%)	1 (33.3%)	13 (31.7%)
American Indian or Alaska Native	3 (14.3%)	1 (14.3%)	0 (0.0%)	0 (0.0%)	0 (0.0%)	0 (0.0%)	0 (0.0%)	4 (9.8%)
Unknown	0 (0.0%)	1 (14.3%)	0 (0.0%)	1 (14.3%)	0 (0.0%)	0 (0.0%)	0 (0.0%)	2 (4.9%)
Diabetes	6 (28.6%)	1 (14.3%)	0 (0.0%)	1 (14.3%)	0 (0.0%)	1 (100.0%)	0 (0.0%)	9 (22.0%)
Untreated HIV	2 (10.5%)	1 (14.3%)	0 (0.0%)	0 (0.0%)	0 (0.0%)	0 (0.0%)	0 (0.0%)	3 (8.1%)
HIV with CD4 ≥ 200 cells/mm^3^	3 (16.7%)	0 (0.0%)	0 (0.0%)	0 (0.0%)	0 (0.0%)	0 (0.0%)	0 (0.0%)	3 (8.3%)
HIV with CD4 < 200 cells/mm^3^	6 (31.6%)	2 (28.6%)	0 (0.0%)	0 (0.0%)	0 (0.0%)	0 (0.0%)	0 (0.0%)	8 (21.6%)
Active cancer	1 (4.8%)	1 (14.3%)	0 (0.0%)	7 (100.0%)	1 (100.0%)	1 (100.0%)	0 (0.0%)	11 (26.8%)
Chemotherapy	1 (4.8%)	1 (14.3%)	0 (0.0%)	3 (42.9%)	1 (100.0%)	1 (100.0%)	0 (0.0%)	7 (17.1%)
Immunotherapy	1 (4.8%)	0 (0.0%)	0 (0.0%)	0 (0.0%)	1 (100.0%)	1 (100.0%)	0 (0.0%)	3 (7.3%)
Prednisone >20 mg/day	1 (4.8%)	0 (0.0%)	0 (0.0%)	1 (14.3%)	0 (0.0%)	0 (0.0%)	0 (0.0%)	2 (4.9%)
Cirrhosis	4 (19.0%)	0 (0.0%)	1 (100%)	0 (0.0%)	0 (0.0%)	0 (0.0%)	0 (0.0%)	5 (12.8%)
Solid organ transplant status	1 (4.8%)	0 (0.0%)	0 (0.0%)	0 (0.0%)	0 (0.0%)	0 (0.0%)	1 (33.3%)	2 (4.9%)
Bone marrow transplant status	0 (0.0%)	0 (0.0%)	0 (0.0%)	0 (0.0%)	0 (0.0%)	0 (0.0%)	0 (0.0%)	0 (0.0%)

**Table 2:** Summary of logistic regression models.

	β-D-Glucan OR (95% CI)	Eosinophil Count OR (95% CI)	AUC (95% CI)	Prediction Accuracy
Model 1	1.006 (1.000, 1.012)		0.786 (0.555, 0.989)	75%
Model 2		108.3 (0.2, 67,707.2)	0.757 (0.563, 0.923)	56%
Model 3	1.012 (0.991, 1.034)	17.9 (0.05, 7089.6)	0.898 (0.716, 1.000)	73.3%

**Table 3:** Association between cocci and an elevated eosinophil count.

	EOS < 0.5	EOS ≥ 0.5
Other	10	0
Cocci	9	6

## Data Availability

Data are contained within the article.

## Supplementary Materials

The following are available online at https://www.mdpi.com/article/10.3390/jof10010029/s1. Table S1. Coccidioidal and Mycobacterial Diagnostics for Final Diagnoses. Table S2. Patient Characteristics of Sample by Status of β-D-Glucan Levels and Eosinophils (Missing or Observed).

## Abbreviations

Coccidioidomycosis (cocci.), cluster of differentiation 4 (CD4); computed tomography (CT); human immunodeficiency virus (HIV); immunoglobulin G (IgG); immunoglobulin M (IgM); picture archiving and communication systems (PACS); rifampin, isoniazide, pyrazinamide, and ethambutol (RIPE); tuberculosis (TB).
