# Supplementary material for: Examining Miliary Disease Etiology in a Coccidioides-Endemic Center: A Retrospective Cohort Study
Source: J Fungi (Basel). 2023 Dec 31;10(1):29. doi: 10.3390/jof10010029 (PMC10817642; doi:10.3390/jof10010029)
Supplement: Supplementary file 1 [file jof-10-00029-s001.zip › jof-2778458-supplementary.pdf]

Table S1. Coccidioidal and Mycobacterial Diagnostics for Final Diagnoses.

	Coccidioidomycosis (N=21)	TB (N=7)	Coccidioidomycosis and TB (N=1)	Metastatic Solid Tumor (N=7)	Lymphoma (N=1)	Other (N=1)	Unknown (N=3)	Total (N=41)
Coccidioides serologies								
Positive	19 (100%)	0 (0.0%)	1 (100%)	0 (0.0%)	0 (0.0%)	0 (0.0%)	0 (0.0%)	20 (62.5%)
Coccidioides complement fixation titer								
< 1:32	10 (62.5%)	0 (0.0%)	1 (100%)	0 (0.0%)	0 (0.0%)	0 (0.0%)	1 (100.0%)	12 (66.7%)
≥ 1:32	6 (37.5%)	0 (0.0%)	0 (0.0%)	0 (0.0%)	0 (0.0%)	0 (0.0%)	0 (0.0%)	6 (33.3%)
Coccidioides growth in culture								
Yes	12 (80.0%)	0 (0.0%)	0 (0.0%)	0 (0.0%)	0 (0.0%)	0 (0.0%)	0 (0.0%)	12 (52.2%)
Coccidioides yielded from pathology specimen								
Yes	6 (85.7%)	0 (0.0%)	0 (0.0%)	0 (0.0%)	0 (0.0%)	0 (0.0%)	0 (0.0%)	6 (54.5%)
Quantiferon Gold for TB								
Positive	0 (0.0%)	5 (100.0%)	1 (100%)	0 (0.0%)	0 (0.0%)	0 (0.0%)	0 (0.0%)	6 (25.0%)
Negative	7 (58.3%)	0 (0.0%)	0 (0.0%)	2 (100.0%)	1 (100.0%)	1 (100.0%)	2 (100.0%)	13 (54.2%)
Indeterminate	5 (41.7%)	0 (0.0%)	0 (0.0%)	0 (0.0%)	0 (0.0%)	0 (0.0%)	0 (0.0%)	5 (20.8%)
Acid-fast bacilli smear								
Positive	0 (0.0%)	2 (40.0%)	1 (100%)	0 (0.0%)	0 (0.0%)	1 (100.0%)	0 (0.0%)	4 (15.4%)
Negative	15 (100.0%)	3 (60.0%)	0 (0.0%)	2 (100.0%)	1 (100.0%)	0 (0.0%)	1 (100.0%)	22 (84.6%)
Acid-fast bacilli culture								
TB	0 (0.0%)	4 (66.7%)	1 (100%)	0 (0.0%)	0 (0.0%)	0 (0.0%)	0 (0.0%)	5 (18.5%)
Nontuberculous mycobacteria	1 (6.7%)	0 (0.0%)	0 (0.0%)	0 (0.0%)	0 (0.0%)	1 (100.0%)	0 (0.0%)	2 (7.4%)
No growth	14 (93.3%)	2 (33.3%)	0 (0.0%)	2 (100.0%)	1 (100.0%)	0 (0.0%)	1 (100.0%)	20 (74.1%)
Nontuberculous mycobacteria subtypes								
Mycobacterium avium complex	1 (100.0%)	N/A	N/A	0 (0.0%)	0 (0.0%)	0 (0.0%)	0 (0.0%)	1 (50.0%)
Mycobacterium simiae	0 (0.0%)	N/A	N/A	0 (0.0%)	0 (0.0%)	1 (100.0%)	0 (0.0%)	1 (50.0%)
TB found on pathology								
Yes	0 (0.0%)	1 (33.3%)	0 (0.0%)	0 (0.0%)	0 (0.0%)	0 (0.0%)	0 (0.0%)	1 (11.1%)
TB polymerase chain reaction testing								
Positive	0 (0.0%)	2 (66.7%)	0 (0.0%)	0 (0.0%)	0 (0.0%)	0 (0.0%)	0 (0.0%)	2 (40.0%)

Table S2. Patient Characteristics of Sample by Status of β-D-Glucan Levels and Eosinophils (Missing or Observed).

	**Missing β-D-Glucan Level, Eosinophils or Both (N=26)**	**Observed β-D-Glucan and Eosinophils (N=15)**	**Total (N=41)**
Female	7 (27%)	5 (33%)	12 (29%)
Age			
Mean (SD)	51.46 (15.02)	50.73 (16.52)	51.20 (15.38)
Race/Ethnicity			
Non-Hispanic White	11 (42%)	6 (40%)	17 (41%)
Black	0 (0%)	1 (7%)	1 (2%)
Asian or Pacific Islander	3 (12%)	1 (7%)	4 (10%)
LatinX	6 (23%)	7 (47%)	13 (32%)
Native American	4 (15%)	0 (0%)	4 (10%)
Not Specified	2 (8%)	0 (0%)	2 (5%)
Diabetes	5 (19%)	4 (27%)	9 (22%)
Active Cancer	7 (27%)	4 (27%)	11 (27%)
Diagnosis			
Coccidioidomyco sis	10 (38%)	11 (73%)	21 (51%)
TB	6 (23%)	2 (13%)	8 (20%)
Metastatic Solid Tumor	6 (23%)	1 (7%)	7 (17%)
Lymphoma	1 (4%)	0 (0%)	1 (2%)
Other	0 (0%)	1 (7%)	1 (2%)
Unknown	3 (12%)	0 (0%)	3 (7%)s
